# Metabolic Syndrome Programming and Reprogramming: Mechanistic Aspects of Oxidative Stress

**DOI:** 10.3390/antiox11112108

**Published:** 2022-10-26

**Authors:** You-Lin Tain, Chien-Ning Hsu

**Affiliations:** 1 Department of Pediatrics, Kaohsiung Chang Gung Memorial Hospital, Kaohsiung 833, Taiwan; 2 College of Medicine, Chang Gung University, Taoyuan 333, Taiwan; 3 Department of Pharmacy, Kaohsiung Chang Gung Memorial Hospital, Kaohsiung 833, Taiwan; 4 School of Pharmacy, Kaohsiung Medical University, Kaohsiung 807, Taiwan

**Keywords:** oxidative stress, reactive oxygen species, antioxidant, developmental origins of health and disease (DOHaD), nitric oxide, obesity, hypertension, metabolic syndrome

## Abstract

Metabolic syndrome (MetS) is a worldwide public health issue characterized by a set of risk factors for cardiovascular disease. MetS can originate in early life by developmental programming. Increasing evidence suggests that oxidative stress, which is characterized as an imbalance between reactive oxygen species (ROS), nitric oxide (NO), and antioxidant systems, plays a decisive role in MetS programming. Results from human and animal studies indicate that maternal-derived insults induce MetS later in life, accompanied by oxidative stress programming of various organ systems. On the contrary, perinatal use of antioxidants can offset oxidative stress and thereby prevent MetS traits in adult offspring. This review provides an overview of current knowledge about the core mechanisms behind MetS programming, with particular focus on the occurrence of oxidative-stress-related pathogenesis as well as the use of potential oxidative-stress-targeted interventions as a reprogramming strategy to avert MetS of developmental origins. Future clinical studies should provide important proof of concept for the effectiveness of these reprogramming interventions to prevent a MetS epidemic.

## 1. Introduction

Emerging evidence suggests that early life environment may negatively affect long-term health and result in increased risk for developing chronic diseases later in life. In a series of studies, David Barker and his colleagues showed that low birth weight (LBW) is associated with increased rates of heart disease, diabetes, and many other features of metabolic syndrome (MetS) in adult life [1,2,3,4]. Based on these findings, David Barker and colleagues proposed the concept of fetal origins of adult disease [5]. It soon became clear that adverse environmental insults also occur during a critical developmental window that produces long-term alterations in tissue structure or function by what is now called developmental programming [6], as well as predisposition to future illness. These developments led to the emergence of the field known as ‘The Developmental Origins of Health and Disease’ (DOHaD) [7]. Notably, the DOHaD concept also provides a novel way to avert adult disease by reprogramming therapy [8,9], that is, by switching therapy prior to illness onset from adulthood to fetal or fetal life. For that reason, reprogramming can potentially serve as an innovative preventive strategy to reduce the global burden of disease.

Non-communicable diseases (NCDs) are of increasing global concern due to their high mortality rate [10]. Importantly, MetS and associated disorders account for two-thirds of NCD deaths [11]. Also important is the prevalence of MetS, which continues to rise globally because of a lack of specific therapeutic regimens [11]. Based on this, the pursuit of a DOHaD approach that can better understand metabolic programming and develop efficient reprogramming strategies has the potential to reduce global burden of MetS.

MetS is a collection of medical conditions that occur together and that increase risk of cardiovascular disease (CVD) [12]. The main components of MetS comprise insulin resistance, obesity, hypertension, non-alcoholic fatty-liver disease (NAFLD), dyslipidemia, and accumulation of adipose tissue. Although the pathogenesis of MetS is highly complex and not yet clear, increasing evidence suggests that oxidative stress has a decisive role in its manifestations [13].

Oxidative stress is a phenomenon caused by an imbalance in overproduction of deleterious reactive oxygen and nitrogen species (ROS and RNS) that overwhelm the capacity of cellular antioxidant defense [14]. Novel research findings increasingly support the importance of oxidative stress in various components of MetS, including hypertension [15], obesity [16], insulin resistance [17], NAFLD [18], etc. Conversely, treatment with antioxidants has been suggested to aid in the prevention of MetS-related disorders [19,20,21].

Despite the evidence showing the impact of oxidative stress and antioxidant therapy in MetS, little attention has been paid to their implications for the developmental programming of MetS. The aim of the current review is to map the best available evidence onto the interplay between oxidative stress and developmental programming of MetS. Our review also tends to highlight the common mechanisms behind MetS programming, their interactions with oxidative stress, and the potential of oxidative-stress-targeted therapy as a reprogramming strategy for MetS of developmental origins.

We used the PubMed, Medline, and Embase databases to search studies written in English using the following keywords: “metabolic syndrome”, “hypertension”, “dyslipidemia”, “hyperlipidemia”, “obesity”, “diabetes”, “insulin resistance”, “hyperglycemia”, “developmental programming”, “DOHaD”, “free radicals”, “offspring”, “progeny”, “mother”, “prenatal”, “nitric oxide”, “oxidative stress”, “pregnancy”, “reprogramming”, “reactive oxygen species”, “reactive nitrogen species”, and “antioxidant”. Additional studies were selected based on references from eligible articles. The search was ended by 23 August 2022.

## 2. Current Evidence Supporting the Developmental Origins of MetS

### 2.1. Human Research

Currently, several lines of epidemiological evidence suggest that adverse intrauterine conditions coincide with the risk of developing MetS throughout the lifetime. Existing human studies mainly come from natural history famine birth cohorts. The studies on the Dutch famine showed that pregnant women under famine had children who developed several features of MetS later in life, such as hypertension, dyslipidemia, obesity, and insulin resistance [22,23]. Studies in other famines also support the notion that early-life famine exposure appears to be a risk factor for obesity, hypertension, and coronary heart disease [22,23,24,25]. Also, data from twin studies suggest that LBW is related to an increased risk of adult cardiometabolic disorders [26,27].

In 1989, Barker and colleagues reported that LBW was associated with an increased risk of death from CVD [1]. Likewise, there have been many studies showing an association between LBW and hypertension [28], impaired glucose tolerance [29], and obesity [30] in later life. Much of the observational research on risk factors for MetS traits represent another line of evidence to support developmental origins of MetS. Risk factors now known to have such effects include maternal malnutrition [22,23], maternal obesity [31,32], gestational diabetes [33], maternal smoking [34], environmental toxins [35], maternal stress [36], etc. Finally, postnatal overnutrition is detrimental for infants with LBW who attain “catch-up growth”, being related to obesity and cardiometabolic risks [37,38]. A systematic review summarizing 39 studies revealed that rapid weight gain in infants with LBW was linked to an 80% greater risk for CVDs [39].

A number of hypotheses, such as thrifty phenotype [40], catch-up growth hypothesis [41], and predictive adaptive responses [42] have been developed to explain the epidemiological observations of an association between early life insults and later adult diseases. Despite these human studies supporting a connection between early-life environmental exposure and developmental origins of MetS traits in later life, these clinical studies seem unable to provide molecular mechanisms underlying developmental origins of MetS for the creation of reprogramming interventions. As a result, the consideration of biological plausibility when assessing causality and the creation of potential reprogramming strategies rely heavily upon evidence derived from animal models.

### 2.2. Animal Models

A number of previous studies address the importance of animal models being used to understand MetS programming, and this has been reviewed elsewhere [43,44,45,46]. Bearing in mind the complexity of MetS, developmental origin studies of MetS are mostly conducted using models that display some, but not all, features of MetS in most investigations [43,44,45,46]. Many animal models are derived from a variety of early-life risk factors to elicit certain characteristics of MetS in adult offspring. Similar to human studies, these early-life insults contribute to the developmental origins of MetS, including maternal nutrition imbalance, maternal illness, environmental toxins, maternal stress, medication use, etc. Although rats are the most frequently used animals [43,44,45,46], other species like mice [47], sheep [48], rabbits [49], pigs [50], and non-human primate [51] have also been used for comparisons of major components of MetS development during the lifetime. As we primarily focus on oxidative stress in this review, and for the sake of brevity, we have limited the animal models of oxidative-stress-related MetS with developmental origins; these are discussed in detail in the following section.

## 3. Oxidative-Stress-Related Developmental Origins of MetS

### 3.1. ROS/NO Disequilibrium

Oxidative stress results from a state of disequilibrium in the ROS/NO balance and a limited biological antioxidant capability. Both ROS and RNS are damaging biological molecules [14]. ROS are highly reactive chemicals formed from oxygen, including free radicals such as superoxide anion (O_2_^−^) and hydroxyl anion (OH^−^) as well as non-radical molecules such as hydrogen peroxide (H_2_O_2_). Among them, the superoxide anion radical initiates a cascade of reactions, resulting in the generation of other ROS species.

RNS that bear nitrogen atoms include the nitric oxide radical (NO^−^), the nitrogen dioxide radical (NO2^−^), and peroxynitrite (ONOO^−^). Much of RNS-dependent cytotoxicity resides in peroxynitrite, which is produced by the reaction between NO and superoxide [52]. In contrast, NO physiologically functions as a gasotransmitter, participating in cardiometabolic health at an optimal level [53]. Asymmetric dimethylarginine (ADMA) is an endogenous competitive inhibitor of NOS [54]. High ADMA can uncouple NOS isoenzymes to generate peroxynitrite, further contributing to reduced NO bioavailability and increased oxidative stress [55].

On the other hand, several antioxidants can counteract the harmful effects of ROS/RNS. Superoxide dismutase (SOD), catalase, glutathione peroxidase (GPx), glutathione reductase, etc. are enzymatic antioxidants. There are also quite a few non-enzymatic antioxidants, which include glutathione (GSH) and vitamins [56]. The discrepancy between excessive ROS/RNS and weak endogenous antioxidant defense leads to damaged DNA, lipids, proteins, and cellular structures.

### 3.2. Oxidative Stress and NO Signaling during Pregnancy

During pregnancy, the balance between ROS and antioxidants should be maintained to provide an appropriate environment for the fetus [57]. The physiological generation of ROS positively impacts a variety of developmental processes, ranging from oocyte maturation [58], embryo implantation [59], and placental differentiation [60] to fetal development. The fetus needs oxygen early in pregnancy, but the oxygen consumption differs at different trimesters of pregnancy [61]. Fetal oxygen levels are low during the first trimester. During the second and third trimesters, increasing oxygen needs are in response to rapid fetal weight gain and establishment of fetal–placental circulation [62]. Increased production of ROS occurs because of high consumption of oxygen, enhanced metabolism, and utilization of fatty acids, while abnormal overproduction of ROS disrupts these processes, resulting in compromised pregnancy [57]. Oxidative damage arises due to the failure of defensive antioxidant mechanisms in responding to excessive ROS and RNS [56]. Adverse conditions in pregnancy that are now known to induce oxidative stress include preeclampsia, diabetes, maternal smoking, obesity, and intrauterine growth retardation (IUGR) [63].

NO has a crucial role in governing feto-placental blood flow. Along with the main vasodilator in the placenta, NO is involved in vascular reactivity regulation, placental bed vascular resistance, and angiogenesis [64]. Circulating ADMA levels, an endogenous inhibitor of NOS, are reduced in the first trimester but increase as the gestational age increases [65,66]. In early pregnancy, low ADMA and concomitant high NO may result in hemodynamic adaptation, a greater need of organ perfusion, and uterine relaxation to allow for fetal growth. In contrast, increased ADMA levels in later pregnancy aid in the higher uterine muscle contractile activity that is required for successful delivery [67]. In compromised pregnancies, such as in pre-eclampsia [67], gestational diabetes [68], and maternal undernutrition [69], ADMA levels rise to levels higher than those seen in normal pregnancy. Summarily, imbalances between ROS and ADMA/NO pathway result in oxidative stress, which is a condition that contributes to fetal programming in compromised pregnancies.

### 3.3. Animal Models of Oxidative-Stress-Related Developmental Origins of MetS

Although mounting evidence indicates the pathogenic interrelationship between oxidative stress and MetS [13], there is a relative paucity of information regarding the impact of oxidative stress in early life on offspring MetS traits. Hence, this section mainly covers evidence regarding animal models used to study oxidative-stress-related developmental origins of MetS. These animal models are summarized in Table 1 [57,58,59,60,61,62,63,64,65,66,67,68,69,70,71,72,73,74,75,76,77,78,79,80,81,82,83,84,85,86,87,88,89,90,91,92,93,94,95,96,97,98,99,100,101,102,103,104,105,106,107,108,109,110,111,112,113,114,115,116,117,118]. Since there is a large amount of available information for single components of MetS, for the sake of brevity, we limited our study to those animal models that display at least two of the components of MetS in offspring. Additionally, this review was restricted to rat models to facilitate appropriate comparisons of major features of MetS as they appear throughout a lifetime. In rats, one month of life is equivalent to 3 human years in adulthood [119]. Table 1 lists the timing of offspring outcomes, ranging from one week to one year of age in rats, which corresponds to humans from infancy to middle adulthood.

#### 3.3.1. Maternal-Derived Insults

Various environmental insults have been examined in animal models, including maternal nutritional imbalance [69,70,71,72,73,74,75,76,77,78,79,80,81,82,83,84,85], pregnancy complications [86,87,88,89,90,91,92,93,94], maternal illness [95,96,97,98,99,100,101,102,103,104,105,106], and toxin/chemical exposure [107,108,109,110,111,112,113,114,115,116,117,118]. Maternal nutritional imbalance can induce nutritional programming. Following the observational studies evaluating exposure to severe famine [22,23,24], maternal caloric or protein restriction models have been conducted to mimic malnutrition in pregnant women exposed to severe famine at that time. Adult rat progeny born to dams exposed to 50% caloric restriction develop insulin resistance and hypertension [69,70,71]. 

Similarly, protein restriction (8–9%) during pregnancy and/or lactation leads to offspring hypertension and insulin resistance [72,73]. Offspring MetS traits can also be programmed by maternal overnutrition. A maternal high-fat diet has been commonly used as an animal model for studying MetS of developmental origins [120]. Mother rats receiving a high-fat diet saw an elevation in BP, body weight, blood lipids, and insulin level in their offspring [74,75,76,77]. Likewise, hypertension, abnormal regulation of lipid metabolism, and insulin signaling can be programmed by a maternal high-fructose diet [80,81,82].

Additionally, complications during pregnancy and maternal illness are able to cause MetS programming. Bilateral uterine artery ligation induced maternal uteroplacental insufficiency that led to hypertension, dyslipidemia, and insulin resistance in adult male rat offspring [86,87]. In addition, adult male offspring born to dams exposed to hypoxia developed hypertension, obesity, and insulin resistance [89,90]. Likewise, offspring hypertension and insulin resistance can be induced by maternal inflammation in a lipopolysaccharide (LPS) exposure model or a surgically induced periodontitis model [92,93].

Several components of MetS such as hypertension, obesity, insulin resistance, and dyslipidemia in adult offspring induced by maternal diabetes are also demonstrable in animal models [95,96,97]. Though many models have been used for diabetes research, only streptozotocin (STZ)-induced diabetes has been modeled for MetS of developmental origins [95,96,97]. Both type 1 and type 2 diabetes can be induced by STZ when given to adult [95] or neonate rats [95,96,97]. Previous reports also demonstrated that adult male offspring in a rat model with maternal continuous light exposure had hypertension and insulin resistance [100,101]. Another common pregnancy complication is maternal stress. A developing fetus is prone to being exposed to excessive glucocorticoid due to a stressed pregnancy. Dexamethasone exposure during pregnancy was shown to induce hypertension, obesity, insulin resistance, and liver steatosis in adult male rat offspring [103,104,105].

Moreover, maternal exposures to toxin/chemical have also been associated with the developmental programming of MetS. Several of the studies listed in Table 1 indicated that maternal exposure to di-n-butyl phthalate (DEHP) [108,109] or bisphenol A (BPA) [111,112] can lead to hypertension and insulin resistance in adult rat offspring. Additionally, maternal nicotine administration during lactation was shown to cause hypertension, hyperlipidemia, and steatosis in adult offspring [113,114,115]. Furthermore, administration of 1 g ethanol/kg on gestational days 13 and 14 in mother rats induced MetS programming, resulting in hypertension and insulin resistance in offspring of both sexes by 6 months of age [116,117].

#### 3.3.2. Mechanisms behind Oxidative Stress

Oxidative-stress-mediated mechanisms involved in the pathogenesis of developmental MetS include increased ROS generation enzymes [84,106,113,118], increased ROS [85,88,89,102,110], decreased expression and/or activity of antioxidant enzymes [72,78,99,106,115,118], increased peroxynitrite [70,98,113], increased oxidative damage [69,72,78,79,83,84,88,94,98,111,113,115], and dysregulated ADMA-NO pathway [69,70,83,95,99,103,107,111]. Notably, most studies have focused on the renal and cardiovascular systems: investigators generally paid less attention to oxidative stress programming on other organ systems, such as the brain [84,102,118], spleen [85], liver [104,105,115], and adrenal gland [106].

Over the years, many oxidative stress biomarkers have been proposed, mainly reflecting the assessment of oxidative damage in biological molecules: lipids, proteins, and DNA. Among these, lipid peroxidation biomarkers are the most commonly used. Table 1 shows how several biomarkers of lipid peroxidation have been utilized to determine oxidative damage in different models of programmed MetS, including F_2_-isoprostanes [72,88], malondialdehyde (MDA) [78,84,94,113], thiobarbituric acid reactive substances (TBARS) [99], and 4-hydroxynonenal (4-NHE) [115]. Notably, MetS of developmental origins programmed by different maternal insults accompanies organ-specific lipid peroxidation in the kidney [72,78,88,94], vessels [99,113], brain [84], and liver [115].

Additionally, 8-hydroxydeoxyguanosine (8-OHdG) is a biomarker used to detect oxidized nucleoside of DNA [121]. Several studies support the idea that oxidative stress with increased renal 8-OHdG expression is involved in the pathogenesis of MetS programming in models of caloric restriction [69], high-fat diet [78], high-fructose diet [83], prenatal dexamethasone exposure [107], and prenatal bisphenol A exposure [111]. Another biomarker of oxidative stress is 3-nitrotyrosine (3-NT), which represents the nitration of protein-bound and free tyrosine residues by reactive peroxynitrite molecules [122]. Prior work revealed that increased 3-NT in the vessels [70,113] and kidneys [98] is related to MetS of developmental origins.

Decreased antioxidant capacities can also be involved in oxidative-stress-related MetS programming. Impaired enzymatic and non-enzymatic antioxidant defenses, including SOD [78,99], glutathione peroxidase 1 [106,115,118], catalase [118], and glutathione [72], have been shown in several models of MetS programming.

**Figure 1:**
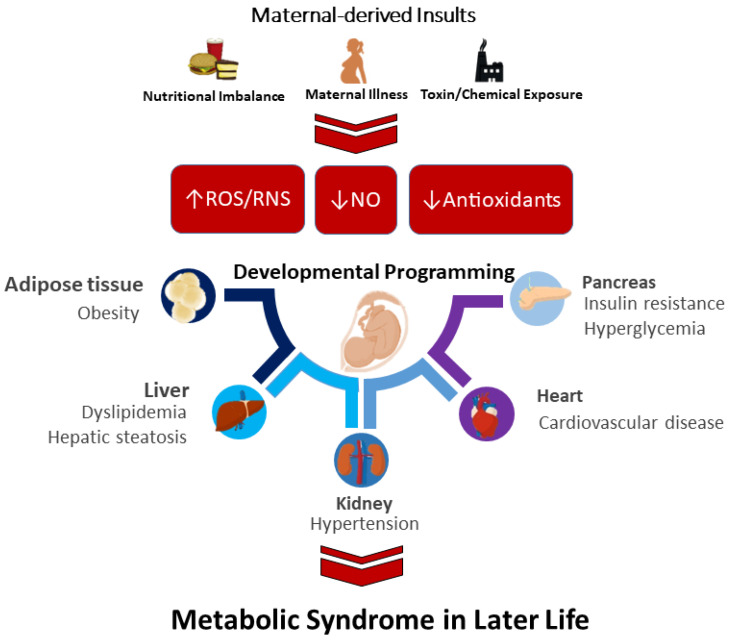
Schema outlining how maternal-derived insults induce MetS in later life via oxidative stress programming of various organ systems. Maternal-dervied insults that induce oxidative stress are related to increases in reactive oxygen/nitrogen species (ROS/RNS), decreases in nitric oxide (NO), and reductions in antioxidants.

Prior reviews support the notion that ADMA-related NO-ROS imbalance in early life induces offspring hypertension, a hallmark of MetS. Table 1 illustrates how ADMA is a key risk factor for oxidative stress programming in several animal models, such as caloric restriction [69], diabetes [95], prenatal dexamethasone exposure [107], and prenatal bisphenol A exposure [111]. Moreover, NO deficiency in the vessels [70,84] and kidneys [69,99,103,111] is also relevant to MetS of developmental origins. A summary of the interaction between maternal-derived insults implicated in oxidative stress and the major organ systems involved in MetS of developmental origins is depicted in Figure 1.

#### 3.3.3. Other Mechanisms Related to MetS Programming

**Figure 2:**
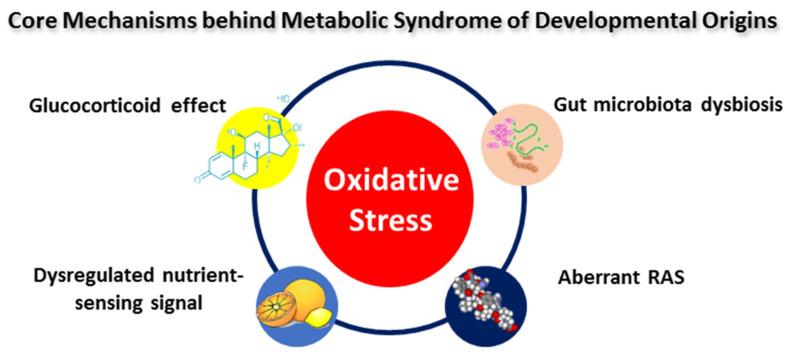
The interconnection between oxidative stress and other common mechanisms underlying metabolic syndromes of developmental origins.

In addition to oxidative stress, several core mechanisms may participate in MetS programming [45], including the glucocorticoid effect [123], dysregulated nutrient-sensing signals [124], aberrant activation of the renin–angiotensin aldosterone system (RAAS) [125], gut microbiota dysbiosis [126], etc. Oxidative stress acts a molecular hub facilitating a wide range of functional interactions among the above-mentioned core mechanisms behind MetS programming (Figure 2). Several of the studies presented in Table 1 have linked maternal glucocorticoid exposure to MetS programming [103,104,105,106,107]. As a product of the activation of the hypothalamic–pituitary–adrenal (HPA) axis, glucocorticoids have potent programming effects on fetal development [127]. Also, the interplay of oxidative stress and nutrient-sensing signals has been implicated in maternal high-fructose diet-induced offspring hypertension [84,128]. Further, it is known that RAAS intrinsic to tissues modulates BP, metabolic homeostasis, adiposity, and insulin sensitivity [125,129]. The aberrant activation of the RAAS and oxidative stress concurrently exist in several models of MetS programming [84,103,130]. Moreover, disruption in gut microbiota is tightly connected to MetS and associated disorders [131], such as obesity [132], insulin resistance [133], dyslipidemia [134], cardiovascular disease [135], etc. An imbalanced redox state induces gut microbiota dysbiosis, while gut microbial communities regulate redox signaling to preserve host–microbiota homeostasis [136,137].

## 4. Reprogramming Strategies: Oxidative-Stress-Targeting Therapies

Although the role of oxidative stress in the pathogenesis of many diseases is undoubted, the beneficial effects of antioxidant therapy, based on available clinical evidence, remain inconclusive. So far, the majority of epidemiological studies have not confirmed any evidence of proven benefits from antioxidant supplementation, especially in the cardiovascular field [138,139]. These controversial findings may be due to the type of antioxidant, the single versus multiple approach, supplement timing and dosage, the population suitable to be treated, etc. Accordingly, it is vital to target specific critical redox pathways and increase the selectivity of these oxidative-stress-targeted approaches in animal models before clinical translation.

As for our contemporary knowledge of the DOHaD concept, it turns out that prevention and management of MetS can be started earlier, even before disease occurs, by reprogramming [8,9]. In the above sections, we illustrated the critical roles that oxidative stress plays in the pathogenesis of MetS programming. On that basis, antioxidants and other oxidative-stress-targeted interventions hold promise for the early-life prevention of MetS in adult progeny.

**Figure 3:**
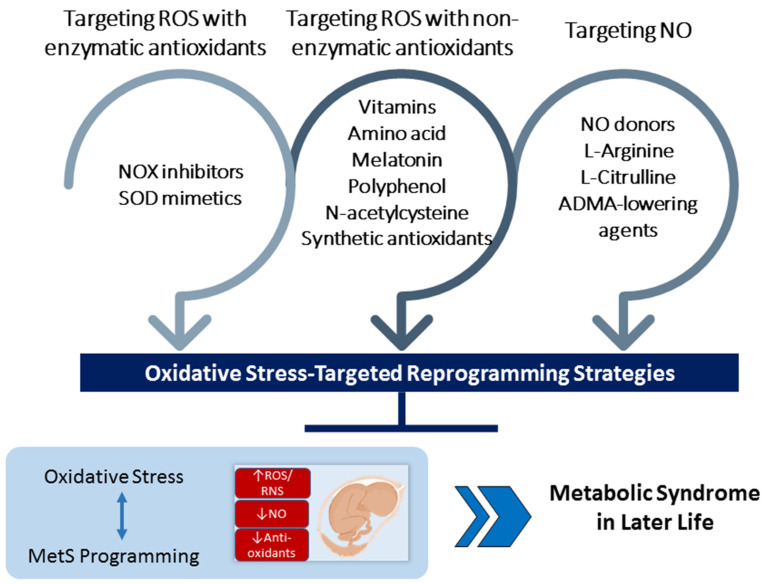
Schema outlining the potential oxidative-stress-targeted interventions as a reprogramming strategy to prevent metabolic syndrome of developmental origins.

Non-enzymatic antioxidants could be natural and synthetic antioxidants [140]. Examples of natural non-enzymatic antioxidants are glutathione, polyphenols, carotenoids, flavonoids, vitamins A, C, and E, etc. [141]. Apart from natural antioxidants, several synthetic antioxidants have also been implemented in MetS. This section discusses the reprogramming role of oxidative-stress-targeted therapies that are involved in the main redox reactions and avert MetS of developmental origins. There are several different types of oxidative-stress-targeted intervention. These are grouped together, depending on which mechanism of oxidative stress they mediate. Overall, these interventions can be classified as targeting ROS with enzymatic antioxidants, targeting ROS with non-enzymatic antioxidants, and targeting NO. These potential oxidative-stress-targeted interventions used as reprogramming therapies for MetS of developmental origins are illustrated in Figure 3.

### 4.1. Targeting ROS with Enzymatic Antioxidants

The NOX family, as a key enzymatic source of ROS, can employ NADPH as an electron donor and then drive molecular oxygen to convert into superoxide [142]. Therefore, agents that would efficaciously target NOXs to scavenge ROS might hold significant promise for reducing oxidative stress [143]. There are two types of NOXs inhibitors: small-molecule inhibitors and peptidic inhibitors [143]. However, neither of them have been examined in MetS of developmental origins.

On the other hand, SOD can eliminate superoxides with a dismutation mechanism. SOD mimetics have also been explored as a potential treatment for many oxidative-stress-related disorders [144]. It has long been known that SOD modulates metabolism. Prior work indicates that several types of SOD mimics show therapeutic potential against dyslipidemia [145], obesity [146], insulin resistance [146], and hypertension [147]. Although administration of SOD mimetic tempol in pregnancy has been reported to reduce BP in spontaneously hypertensive rat offspring [148], none of the SOD mimetics have been approved in models of MetS programming to date.

### 4.2. Targeting ROS with Non-Enzymatic Antioxidants

Several non-enzymatic antioxidants applied during gestation and lactation have been utilized as reprogramming strategies to prevent the development of MetS in animal models, including vitamins, amino acids, melatonin, polyphenol, N-acetylcysteine (NAC), and synthetic antioxidants.

#### 4.2.1. Vitamins

The most widely explored nutraceuticals are vitamins C and E. Vitamin C is a potent water-soluble antioxidant with the ability to quench ROS [149]. Vitamin E is a lipid-soluble antioxidant that inhibits NADPH oxidase, cyclooxygenase, and lipoxygenase [150]. Our prior review summarizes current evidence supporting perinatal use of vitamins C and E, alone or combined with other antioxidants, for protecting rat offspring hypertension [151]. Disruption of epigenetic regulation can result in oxidative stress in relation to MetS programming [152]. Despite a recognized role of vitamins B6, B12, and folate as methyl donors for DNA methylation [153], whether their supplementations in pregnancy can avert offspring MetS via regulation of epigenetics remain largely unknown. Although several vitamins exert advantageous effects on oxidative-stress-related disorders, less attention has been paid to determine their reprogramming effects on MetS of developmental origins.

#### 4.2.2. Amino Acids

Several amino acids have antioxidant properties [154]. It is well-known that amino acids participate in body fat composition [155], insulin signaling [155], and BP regulation [156]. Previous research indicates that amino acid supplementation during gestation and lactation can avert offspring hypertension in several animal models. Examples of amino acids are taurine, arginine, citrulline, cysteine, and branched-chain amino acids (BCAAs). BCAA supplementation in pregnancy does not only prevent maternal caloric-restriction-induced offspring hypertension [157]: gestational supplementation of BCAAs also benefits obesity-associated insulin resistance programmed by maternal high-fat diet [158].

Even though there are other amino acids showing reprogramming potential for hypertension of developmental origins [156], their reprogramming effects in other MetS traits remain largely unclear. Importantly, amino acid metabolism between the mother and the fetus in pregnancy is crucial for fetal development. We must elucidate the pathophysiologic roles of specific amino acids and their connections in the developmental programming of MetS to avoid unintentional adverse consequences.

#### 4.2.3. Melatonin

Melatonin (N-acetyl-5-methoxytryptamine) is a pleiotropic hormone essential for pregnancy and fetal development [159]. Melatonin and its metabolites, acting as naturally occurring antioxidants, can scavenge ROS/RNS, enhance expression of antioxidant enzymes, and increase NO bioavailability [160,161]. Perinatal use of melatonin has been proposed as a reprogramming strategy for many DOHaD-related adult diseases [162].

As shown in Table 1, the beneficial effects of maternal melatonin therapy are expressed in different models against offspring hypertension [100], insulin resistance [101], and liver steatosis [106]. Perinatal use of melatonin can have beneficial effects against rat offspring hypertension via restoration of the ROS/NO balance in a maternal caloric restriction model [163] and a high-fructose model [164]. Additionally, prior studies have demonstrated interplay between melatonin and several core mechanisms underlying MetS programming, such as aberrant RAAS, dysregulated nutrient-sensing signaling, and glucocorticoid programming [161]. These observations support the notion that perinatal use of melatonin may act in diverse ways to avert MetS programming-induced disorders in later life [161]. Melatonin is also involved in epigenetic regulation [165]. Melatonin can regulate antioxidant and pro-inflammatory genes via epigenetic on/off mechanisms [166]. While maternal melatonin therapy can epigenetically alter more than 450 transcripts in the 1-week-old offspring kidney [165], whether epigenetic regulation of melatonin has a role in its protective effect in MetS programming remains to be elucidated.

Of note is that melatonin is a quite safe supplement in humans [167]. Although the clinical use of melatonin during pregnancy remains inconclusive, it has nonetheless been clinically used for several neonatal diseases [168]. Therefore, there is a desperate need for further translational research into the long-term MetS-associated outcomes of perinatal melatonin use.

#### 4.2.4. Polyphenols

Polyphenols are the widespread phytochemical antioxidants in food [169]. Prior work has revealed the valuable effect of polyphenols in the counterbalance of oxidative stress by working as free-radical scavengers, NOS activators, metal chelators, and stimulator of antioxidant enzymes [170,171]. Accordingly, polyphenols have shown beneficial effects in MetS [172,173]. Though several systematic reviews have shown that dietary polyphenol intake reduces CVD risk [174,175,176,177], only a few polyphenols have been tested in animal models of MetS programming.

Polyphenols are commonly categorized as flavonoids and nonflavonoids [169]. Several flavonoids are potent antioxidants [169]. As an antioxidant, quercetin has been used in pregnancy to protect adult rat progeny against hypertension programmed by maternal high-fat diet [106]. In another antenatal dexamethasone exposure rat model, maternal treatment with epigallocatechin gallate moderated the developmental programming of hypertension [106].

Resveratrol is a nonflavonoid polyphenol that is commonly used as a nutritional supplement [170,178]. Resveratrol can act as an antioxidant against oxidative stress. Currently, there is accumulating evidence that suggests a reprogramming effect of resveratrol for the prevention of offspring MetS [179]. The use of resveratrol in early life has been reported to protect rat offspring against hypertension [107,111], hyperlipidemia [75], obesity [76,180], and insulin resistance [181] in various developmental programming models.

Also, genistein, curcumin, and resveratrol have been demonstrated to trigger the antioxidant and anti-inflammatory machinery and ameliorate MetS traits via epigenetic mechanisms [182]. However, further research is needed to understand whether the beneficial effects of polyphenols in MetS programming are directly related to epigenetic changes [183].

One major issue that limits the clinical translation of polyphenols is their low bioavailability in vivo [184]. Considering the complexity and inter-individual variability of polyphenol pharmacokinetics, further research is required to better elucidate the differential impact of various polyphenols on the MetS of developmental origins.

#### 4.2.5. N-acetylcysteine

N-acetylcysteine, an antioxidant naturally found in Allium plant, is a precursor to glutathione [185]. Also, NAC is a stable L-cysteine analogue and can be used for H_2_S synthesis [186]. Perinatal NAC therapy averts rat offspring hypertension as induced by a number of early-life insults, such as maternal nicotine exposure [114], maternal hypertension [187], maternal L-NAME exposure [188], suramin-induced pre-eclampsia [189], and prenatal dexamethasone and postnatal high-fat diet [190].

Using a maternal L-NAME exposure model, perinatal NAC therapy was shown to protect rat offspring hypertension, accompanied by enhancement of H_2_S-generating enzyme expression and activity in offspring kidneys [188]. In another study [190], the advantageous effects of NAC against offspring hypertension were associated with an increase in plasma glutathione level, reduction of oxidative stress, and upregulation of H_2_S-generating enzymes. Furthermore, maternal NAC therapy was able to avert rat offspring hypertension programmed by maternal suramin administration, which coincided with increased glutathione levels, restoration of NO bioavailability, and augmentation of H_2_S pathways [189].

#### 4.2.6. Synthetic Antioxidants

In addition to natural antioxidants, some synthetic antioxidants have been applied to reduce oxidative stress in animal models to study MetS programming. The transcription factor NRF2 is a master regulator of various homeostatic genes that defend against oxidative stress [191]. In response to oxidative stress, NRF2 is released from its principal negative regulator Kelch-like ECH-associated protein 1 (KEAP1) and translocated to the nucleus, where NRF2 promotes the expression of several antioxidant genes via binding to antioxidant response element (ARE) [192]. Accordingly, NRF2 activators are considered as potential agents to protect oxidative-stress-related damage [193].

Dimethyl fumarate (DMF), an NRF2 activator, has been used to prevent rat offspring hypertension in a combined maternal dexamethasone exposure and postnatal high-fat diet model [194,195]. In addition, maternal lazaroid therapy, an inhibitor of lipid peroxidation [196], prevented the elevation of BP in adult rat progeny born to dams that received a protein-restricted diet [72]. Although certain synthetic antioxidants have been explored in several animal models of oxidative stress, little is known regarding their ability to protect adult offspring against MetS programming.

### 4.3. Targeting NO

A number of NO-targeted approaches have been utilized to increase NO bioavailability, such as NO donors, supplementation of NO substrate, enhancement of the expression and/or activity of NOS, ADMA-lowering agents, etc. So far, some of them have been examined for therapeutic prevention of MetS programming.

While NO donors, molsidomine, and pentaerythritol tetranitrate have shown beneficial effects against the development of hypertension [197,198], their reprogramming effects on MetS traits deserve further clarification.

As the substrate for NOS isoenzymes, L-arginine supplementation has been applied to augment NO bioavailability in several diseases [199], while the beneficial effects of L-arginine from human trials remain inconclusive [200]. As the main precursor of L-arginine, oral l-citrulline supplementation has been utilized to increase l-arginine production and bypass hepatic metabolism to raise NO levels [201]. To date, gestational L-citrulline supplementation has shown benefits against offspring hypertension in rat models of maternal caloric restriction [69], streptozotocin-induced diabetes [95], and prenatal dexamethasone exposure [103]. Along with averting hypertension, L-citrulline supplementation has also attenuated liver fat accumulation and prevented hypertriglyceridemia in adult rat offspring born to dams that received a high-fructose diet [202].

The use of ADMA-lowering agents is another way to increase NO. Though a specific ADMA-lowering agent remains inaccessible at the time of this paper, a number of clinically used drugs have been shown to restore ROS/NO balance throughout lowering ADMA levels [203]. Telmisartan, glucagon-like peptide-1 receptor agonist, rosuvastatin, and epigallocatechin-3-gallate can lower ADMA levels via reduced expression of ADMA-generating enzyme. On the other hand, metformin, NAC, melatonin, atorvastatin, salvianolic acid A, telmisartan, oxymatrine, and rosuvastatin can augment the activity and/or expression of ADMA-metabolizing enzymes and thus decrease ADMA levels [203]. So far, only a few ADMA-lowering agents have been studied in developmental programming models to prevent offspring hypertension, including NAC [191], melatonin [204], and metformin [205]. Metformin also showed benefits against liver steatosis in a maternal high-fat diet rat model [206]. Moreover, supplementing melinjo (Gnetum gnemon) seed extract during lactation protected adult female rat offspring hypertension by enhancing eNOS expression in a maternal high-fructose diet model [207].

### 4.4. Pros and Cons

Although animal studies implicate oxidative stress as an attractive target for MetS prevention and therapy, their efficacy still awaits validation in human trials. Considering the difficulties of recruiting pregnant or lactating women in medication research, the use of breastmilk as a reprogramming strategy would be a good start. Breastmilk has a powerful antioxidant composition [208]. There are reports that suggest that there is a relationship between premature infants fed with breastmilk and lower rates of MetS in young adult life [209]. As breastfeeding is recommended for infants during the first 6 months after birth [210], the antioxidant protection offered by breastfeeding against MetS programming is a key issue that deserve further study.

On the other hand, oxidative-stress-targeted therapy can also be disadvantageous. Most oxidative-stress-targeted therapies such as antioxidants are administered orally or intravenously, which eventually enter the circulation and reach the targeted organ. However, healthy tissues other than the targeted organs, which have not experienced oxidative stress damage, may be non-specifically targeted by the antioxidant [211]. As a result, healthy tissues/organs may be affected negatively, as their levels of ROS may fall below their physiologically normal limit. As homeostasis of ROS is one of the mandatory requirements for normal pregnancy and fetal development [57], antioxidant supplementation during pregnancy and breastfeeding would only apply in the case of deficits, but not as a usual dietary supplement.

Moreover, excessive antioxidant supplement may shift oxidative stress to an opposite state, namely antioxidant stress [212]. However, it currently remains unclear which pathogenetic mechanism should be targeted, which timing for reprogramming should be appropriate, and which kind of antioxidants should be used. Further studies are required to establish the particular developmental window (e.g., prenatal or pre-weaning stage), to elucidate organ-specific redox-sensitive signaling responsible for different maternal-derived insults underlying MetS programming, and to determine the ‘right’ oxidative-stress-targeted therapy with the ‘right’ dose at the ‘right’ time for reprogramming.

## 5. Concluding Remarks and Perspectives

There is substantial evidence that suggests that oxidative stress is involved in MetS programming, and oxidative-stress-targeted therapy is a potential preventive strategy. Our review highlights how targeting ROS with enzymatic antioxidants, targeting ROS with non-enzymatic antioxidants, and targeting NO might represent promising tools for the prevention of MetS and associated disorders. However, with all the obvious benefits of oxidative-stress-targeted therapy in MetS programming, we have to be mindful of timing, dosage, and target organ for various pathologies, since the heterogeneity of MetS has to be a central consideration. Although several oxidative-stress-targeted strategies were explored in animal studies and some of them revealed promising data, their efficacy still awaits future translation into human investigations.

While there has been significant progress in establishing animal models for studying MetS of developmental origins, only a few models present all components of MetS. While some oxidative-stress-targeted therapies offer substantial progress in certain characteristics of MetS, it remains unclear whether their effects are beneficial for other MetS traits or if they should be translated from one model into other models. Importantly, MetS of developmental origins, along with oxidative stress, is associated with other core mechanisms. Therefore, it remains to be determined whether the protective effects of antioxidant therapy in pregnancy are related to the common mechanisms behind MetS programming.

A deeper understanding of the molecular and biochemical mechanisms of abnormalities associated with oxidative stress in MetS programming will facilitate the development of preventive therapeutics. Such efforts might prove effective in the prevention of a global epidemic of MetS.

## Figures and Tables

**Table 1:** Summary of oxidative-stress-related developmental origins of MetS in rodent animal models.

Animal Models	Timing and Dose	Offspring Species/Gender	MetS-Related Outcomes in Offspring	Mechanisms of Oxidative Stress	Programmed Organ System
Caloric restriction	50% caloric restriction during pregnancy and lactation	SD rats/M [69,70]; Wistar rats/M [71]	Hypertension: 12–16 weeks [69,70]; insulin resistance: 14 weeks [71]	↑ ADMA, ↓ NO, ↑ renal 8-OHdG expression [69]; ↑3-NT, ↓ NO [70]	Kidney [69], vessel [70]
Protein restriction	9% low-protein diet during pregnancy [72]; 8% low-protein diet during pregnancy and lactation [73]	Wistar rats/M [72,73]	Hypertension: 12 weeks [72]; insulin resistance: 12 weeks [73]	↑ F_2_-isoprostane, ↓ glutathione [72]	Kidney [72]
Maternal high-fat diet	58% high-fat diet during pregnancy and lactation [74,75,76,77,78]; 31% high-fat high-cholesterol diet during pregnancy [79]	SD rats/M [74,75,76,77,78]; Wistar rats/M & F [79]	Hypertension: 16 weeks [74]; ↑adiposity: 16 weeks [75]; dyslipidemia: 16 weeks [76]; obesity, dyslipidemia, and hyperinsulinemia: 100 days [77]	↓ SOD activity in M; ↑ renal MDA level in F [78]; ↑ renal 8-OHdG expression [79]	Kidney [78,79]
Maternal high-fructose consumption	60% high-fructose diet during pregnancy and lactation [80,81]; 10% wt/vol fructose solution during pregnancy [82]	SD rats/M [80,81]; C57BL/6J/M & F [82]	Hypertension, insulin resistance, and dyslipidemia: 12 weeks [80,81]; hypertension, insulin resistance, and obesity: 1 year [82]	↑ Renal 8-OHdG expression, ↓ NO [83]; ↑brain NADPH-oxidase expression and MDA [84]; ↑ ROS [85]	Kidney [83], brain [84], spleen [85]
Uteroplacental insufficiency	Bilateral uterine artery ligation on day 18 [86] or 19 [87] of pregnancy	Wistar–Kyoto rats/M [86]; Wistar rats/M [87]	Hypertension: 22 weeks [86]; dyslipidemia and insulin resistance: 30 weeks [87]	↑ Urinary F_2_-isoprostane level & renal NADPH-oxidase-dependent superoxide [88]	Kidney [86,88]
Maternal hypoxia	Hypoxia exposure (13% O_2_) from day 6 to 20 of gestation [89]; alternating cycles of normoxic (room air; 120 s) and hypoxic (6.5% O_2_; 80 s) exposure during pregnancy [90]	Wistar rats/M [89]; SD rats/M [90]	Hypertension: 4 months [89]; obesity and insulin resistance: 12 weeks [90]	↑ Lipid peroxidation [91]	Heart [91]
Maternal inflammation	Intraperitoneally administered 0.79 mg/kg LPS on gestational day 8, 10, and 12 [92]; surgically induced periodontitis 13 days before mating [93]	SD rats/M & F [92]; Wistar rats/M [93]	Hypertension: 12 weeks [92]; insulin resistance: 75 days [93]	↑ Renal MDA [94]	Kidney [94]
Maternal diabetes	Intraperitoneally administered 45 mg/kg STZ on gestational day 0 [95]; intraperitoneally administered 50 mg/kg STZ on postnatal day 1 [95]; intraperitoneally administered 120 mg/kg STZ on postnatal day 5 [96,97]	SD rats/M [95]; Wistar rats/M [96,97]	Hypertension: 12 week [95]; obesity: 12 weeks [96]; insulin resistance and dyslipidemia: 16 weeks [97]	↑ ADMA,↓ NO [95]; ↑ renal TBARS and 3-NT [98]; ↑ ROS,↓ NO,↓ SOD activity [99]	Kidney [95,98], vessel [99]
Maternal chronodisruption	Continuous light exposure during pregnancy and lactation [100]; continuous light exposure from day 12 to 21 of gestation [101]	SD rats/M [100]; Wistar rats/M [101]	Hypertension: 12 weeks [100], insulin resistance: 18 weeks [101]	↑ Brain ROS [102]	Brain [102]
Maternal stress	Intraperitoneally administrated 0.2 mg/kg dexamethasone daily on gestational days 15 and 16 [103]; intraperitoneally administered 0.1 mg/kg dexamethasone from 14 to 20 of gestation [104,105]	SD rats/M [103,104,105]	Hypertension: 16 weeks [103]; obesity, insulin resistance, and hypertension: 6 months [104]; liver steatosis: 1 week [105]	↓ Renal NO [103]; ↑ NADPH-oxidase, ↓ Gpx1 expression [106]; ↑ renal 8-OHdG expression, ↑ ADMA [107]	Kidney [103,106], liver [104,105], adrenal gland [106]
Maternal di-n-butyl phthalate (DEHP) exposure	Oral gavage with 6.25 mg/kg DEHP during pregnancy and lactation [108]; oral gavage with 100 mg/kg DEHP from gestational day 9 to postnatal day 21 [109]	Wistar rats/M [108]; SD rats/M [109]	Hypertension: 21 weeks [108]; insulin resistance: 80 days [109]	↑ Renal ROS [110]	Kidney [110]
Prenatal bisphenol A (BPA) exposure	Oral gavage with 50 μg/kg BPA during pregnancy and lactation [111]; oral 240 μg/kg BPA from 2 weeks prior to mating and through pregnancy and lactation [112]	SD rats/M [111]; SD rats/M & F [112]	Hypeertension: 16 weeks [111]; insulin resistance: 6 months [112]	↑ Renal 8-OHdG expression, ↑ ADMA, ↓ NO [111]	Kidney [111]
Maternal nicotine exposure	Nicotine administration through an osmotic minipump at 4 µg/kg/min from day 4 of pregnancy to postnatal day 10 [113,114]; nicotine administration through an osmotic minipump at 6 mg/kg/day from postnatal days 2 to 16 [115]	SD rats/M [113,114]; Wistar rats/M & F [115]	Hypertension: 5–8 months [113,114]; hyperlipidemia and steatosis: 6 months [115]	↑ 3-NT, MDA, and NADPH oxidase [113]; ↑ MDA and 4-NHE levels, ↓ GPx1 activity [115]	Vessel [113], liver [115]
Maternal ethanol exposure	Oral gavage with 1 g of ethanol/kg on gestational day 13 and 14 [116,117]	SD rats/M & F [116,117]	Hypertension: 6 months [116], insulin resistance: 6 months [117]	↓ SOD1, CAT, and Gpx1; ↑ NOX2 [118]	Brain [118]

SD = Sprague–Dawley rat; M = Male; F = Female; LPS = lipopolysaccharide; STZ = streptozotocin; ADMA = asymmetric dimethylarginine; NO = nitric oxide; 8-OHdG = 8-hydroxy-2′–deoxyguanosine; ROS = reactive oxygen species; TBARS = thiobarbituric acid; 3-NT = 3-nitrotyrosine; 4-NHE = 4-hydroxynonenal; Gpx1 = glutathione peroxidase 1; MDA = malondialdehyde; SOD = superoxidase dismutase; CAT = catalase; NOX2 = NADPH oxidase 2.
